# Non-invasive brain stimulation reorganises effective connectivity during a working memory task in individuals with Neurofibromatosis Type 1

**DOI:** 10.1016/j.ynirp.2025.100258

**Published:** 2025-03-29

**Authors:** Marta Czime Litwińczuk, Shruti Garg, Stephen R. Williams, Jonathan Green, Caroline Lea-Carnall, Nelson J. Trujillo-Barreto

**Affiliations:** a School of Health Sciences, University of Manchester, Manchester, United Kingdom; b Division of Informatics, Imaging and Data Science, Faculty of Biology, Medicine and Health, University of Manchester, Manchester, United Kingdom; c Geoffrey Jefferson Brain Research Centre, Manchester Academic Health Science Centre, Manchester, United Kingdom; d School of Psychology, Manchester Metropolitan University, Manchester, United Kingdom

**Keywords:** Neurofibromatosis, Non-invasive brain stimulation, tDCS, Effective connectivity, Working memory, GABA, Magnetic resonance spectroscopy

## Abstract

**Introduction:**

In a previous study, we examined the effect of atDCS on working memory task performance and modulation of the inhibitory neurotransmitter, gamma-aminobutyric acid (GABA), in the dorsolateral prefrontal cortex (dlPFC). The present study investigates whether tDCS modulates effective connectivity during the task, specifically assessing whether tDCS alters interactions between neuronal populations.

**Methods:**

Eighteen adolescents with Neurofibromatosis Type 1 (NF1) completed a single-blind sham-controlled cross-over randomised tDCS trial (with the anode at F3 and cathode at Cz). Dynamic causal modelling was used to estimate the effective connectivity between regions that showed working memory effects from the fMRI. Group-level inferences for between sessions (pre- and post-stimulation) and stimulation type (tDCS and sham) effects were carried out using the parametric empirical Bayes approach. A correlation analysis was performed to relate the estimated effective connectivity parameters of left dlPFC pre-tDCS and post-tDCS to the concentration of GABA measured via magnetic resonance spectroscopy (MRS-GABA). Next, correlation analysis was repeated using all working memory performance and all pre-tDCS and post-tDCS connectivity parameters.

**Results:**

It was found that tDCS decreased average excitatory connectivity from dlPFC to left superior frontal gyrus and increased average excitatory connectivity to left globus pallidus. Further, reduced average intrinsic (inhibitory) connectivity of left dlPFC was associated with lower MRS-GABA. However, none of the connectivity parameters of dlPFC showed any association with performance on a working memory task.

**Conclusions:**

These findings suggest that tDCS reorganised connectivity from frontal to fronto-striatal connectivity. As tDCS-related changes were not specific to the effect of working memory, they may have impacted general cognitive control processes. In addition, by reducing MRS-GABA, tDCS might make dlPFC more sensitive and responsive to external stimulation, such as performance of cognitive tasks.

## Highlights

• AtDCS was applied to left dlPFC in NF1 patients during working memory.
• After atDCS, no effect on modulatory connectivity.
• Evidence for increased N-back average connectivity from dlPFC to globus pallidus.
• Less dlPFC MRS-GABA was associated with less dlPFC inhibition.

## 1 Introduction

Neurofibromatosis 1 (NF1) is a single-gene autosomal dominant neurodevelopmental disorder with birth incidence of 1:2700 Evans et al. (2010). The condition is caused by mutation of the NF1 gene, encodes the neurofibromin protein, and regulates the Ras-MAPK molecular pathway (Daston and Ratner, 2005; North et al., 1997). Physiologically, NF1 is associated with skeletal abnormalities, brain and peripheral nerve tumours (Gutmann et al., 2017). In addition, NF1 patients tend to underperform in academic settings compared to their siblings without an NF1 diagnosis, including poorer performance on assessments of intelligence, visuospatial skills, social competence, executive function and attention (Lehtonen et al., 2012). Notably, Shilyansky et al. (2010) found that NF1 patients score lower on working memory tasks than controls and that they are more sensitive to the effects of increased memory load than controls. In addition, Shilyansky et al. (2010) explored the neural substrates of these deficits using functional magnetic resonance imaging (fMRI) and demonstrated that deficits in working memory of NF1 patients were associated with hypoactivity in dorsolateral prefrontal cortex (dlPFC), frontal eye fields, and parietal cortex, as well as striatal regions. Thus, the working memory deficits in NF1 patients may be explained by disrupted activity of left frontal regions associated with working memory.

To address these physiological and psychosocial alterations, clinical neuroscience has explored therapeutic effects of non-invasive brain stimulation (NIBS). Application of NIBS has shown some success in improving cognitive functioning in children and adolescents with neurodevelopmental conditions, such as autism spectrum condition and attention deficit hyperactivity disorder (Demirtas-Tatlidede et al., 2013; Finisguerra et al., 2019; Westwood et al., 2021). In a previous study, Garg et al. (2022) investigated effects of NIBS on working memory performance in NF1 patients. Transcranial direct current stimulation (tDCS) was applied to left dlPFC during performance of a working memory task (Barbey et al., 2013; Kane and Engle, 2002). F3-Cz tDCS (with the anode at F3) was hypothesized by to increase cortical excitability through depolarisation of the resting membrane potential. Garg et al. (2022) demonstrated that application of tDCS was associated with decreased levels of GABA in the dlPFC, as measured with magnetic resonance spectroscopy (MRS). However, tDCS had transient effects on blood oxygen level dependent (BOLD) signal in fMRI data, and it did not influence behavioural outcomes.

While the original study by Garg et al. (2022) investigated whether tDCS caused changes to local neurotransmitter levels and local BOLD activity, the present work addresses a gap in knowledge by examining how tDCS influences effective connectivity. Specifically, we explore how brain regions coordinate their activity during working memory tasks before and after NIBS. To address this, we conducted a further analysis of the data obtained by Garg et al. (2022), focusing on the effects of a sham-controlled F3-Cz tDCS trial on effective connectivity of the left dlPFC in adolescent participants with NF1. This work will employ dynamic causal modelling (DCM) (Friston et al., 2003). Since in the original Garg et al. (2022) study the administration of tDCS aimed to increase dlPFC excitability, we hypothesise that tDCS will reduce inhibitory intrinsic connectivity (self-connectivity) of left dlPFC, which will increase connectivity from left dlPFC to other regions involved with working memory. We further hypothesise that effective connectivity parameters of left dlPFC will show a positive correlation with GABA concentration and working memory performance.

## 2 Methods

### 2.1 NF1 participants

The data used in this study has been previously described in Garg et al. (2022). Thirty-one adolescents (16 males, 15 females) aged 11–17 years were recruited via the Northern UK NF- National Institute of Health diagnostic criteria [National Institutes of Health Consensus Development Conference. Neurofibromatosis conference statement. Arch. Neurol. 45, 575–578 (1988).] and/or molecular diagnosis of NF1; (ii) No history of intracranial pathology other than asymptomatic optic pathway or other asymptomatic and untreated NF1-associated white matter lesion or glioma; (iii) No history of epilepsy or any major mental illness; (iv) No MRI contraindications. Participants on pre-existing medications such as stimulants, melatonin or selective serotonin re-uptake inhibitors were not excluded from participation. The study was conducted in accordance with local ethics committee approval (Ethics reference: 18/NW/0762, ClinicalTrials.gov Identifier: NCT0499142. Registered August 5, 2021; retrospectively registered, https://clinicaltrials.gov/ct2/show/NCT04991428). All methods were carried out in accordance with relevant guidelines and regulations.

### 2.2 Study design

**Fig. 1:**
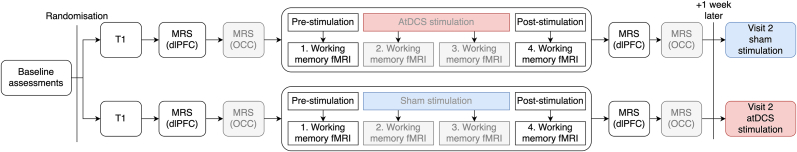
A schematic illustration of the study design and image acquisition. Depending on randomisation, during Visit 1, either atDCS or sham stimulation was applied during session 2 and 3 of working memory fMRI acquisition.

The impact of tDCS on working memory was assessed through a two-arm, single-blind (participant), sham-controlled cross-over study (Fig. 1). Each participant attended two visits, spaced at least one week apart. During each visit they received either the tDCS or sham stimulation, with the order randomized and counterbalanced. Participants maintained their regular medication schedules, including stimulants. During the visits, participants were comfortably positioned in a scanner, and high-resolution T1-weighted images were acquired. Participants performed the N-back working memory task during four 6-min-long sessions (24 min in total) while fMRI data were collected. Stimulation (tDCS or sham) was applied for 15 min during sessions 2 and 3. Between each session, the participants were asked if they were comfortable, and instructions were repeated. Additionally, T2-weighted images were acquired during the first visit and evaluated by a paediatric neuroradiologist to exclude NF1-associated tumours.

### 2.3 Working memory task

The N-back task was used to assess working memory performance in the participants (Kirchner, 1958). Within the scanner, participants were presented with a sequence of black coloured letters on a white screen. The participants were instructed to respond only to the target by pressing a handheld button. During the 0-back condition, the participants responded when the letter ‘X’ was presented on the screen. During the 2-back condition, the participants responded when letter on the screen matched the letter 2 screens before. The stimuli were presented for 2.5 s. Each session consisted of 6 blocks of 0-back condition and 6 blocks of 2-back condition, each block was 30 s long and consisted of 9 target stimuli. Accuracy was calculated separately for 0-back and 2-back conditions (correct hits + correct omissions/total responses). Response times (RT) were calculated only for time to correct response to target stimuli. Inverse efficiency score (IES) was calculated by dividing RT by accuracy as a measure of speed accuracy trade-off, in which lower scores are generally associated with better cognitive performance (faster response at less accuracy cost) (Bruyer and Brysbaert, 2011).

### 2.4 Stimulation

Stimulation was administered using a NeuroConn DC-STIMULATOR MR, with the anode positioned at F3 (electrode size: 5 cm × 5 cm) and the cathode at Cz (electrode size: 5 cm × 7 cm) according to the international 10–20 system. The scalp was first cleaned with Nuprepgel, and Ten20-paste was applied as a conductive medium between the scalp and the electrodes. During tDCS stimulation, the current was gradually increased over 15 s, maintained at 1 mA for 15 min, and then decreased over 15 s. During sham stimulation, the current was ramped up over 15 s and then immediately turned off. The parameters for the current were determined based on prior safety trials conducted with this cohort (clinical trials identifier: NCT03310996).

### 2.5 Structural MRI and MRS acquisition

Scanning was conducted using a Philips Achieva 3 T MRI scanner (Best, NL) equipped with a 32-channel head coil. First, 3D T1-weighted magnetic resonance images were obtained in the sagittal plane with a magnetization-prepared rapid acquisition gradient-echo sequence (repetition time = 8.4 ms; echo time = 3.77 ms; flip angle = 8°; inversion time = 1150 ms; in-plane resolution = 0.94 mm; 150 slices with 1 mm thickness). Next, a T2-weighted structural scan was performed using a turbo spin echo sequence (TR = 3756 ms; TE = 89 ms; 40 slices of 3 mm thickness and 1 mm gap; in-plane resolution = 0.45 mm). Single-voxel ^1^H MRS data were collected before and after stimulation from two volumes of interest (VOI) in each participant: one VOI (40 × 20 × 24 mm) in the left dlPFC and a control VOI (20 × 50 × 20 mm) in the posterior occipital lobe (OCC), centred on the mid-sagittal plane to encompass both hemispheres. Water-unsuppressed spectra were recorded from these locations to serve as references. To detect GABA+ (so called, because the edited signal contains contributions from co-edited macromolecules as well as GABA), water-suppressed GABA-edited MEGA-PRESS spectra (Mescher et al., 1998; Mullins et al., 2014) were acquired (repetition time = 2000 ms; echo time = 68 ms; 1024 sample points at a spectral width of 2 kHz, as detailed in Sanaei Nezhad et al. (2017). The acquisition for the dlPFC MRS took approximately 7 min, with 96 averages, while the occipital voxel MRS took about 3 min, with 32 averages. The number of averages was adjusted to ensure comparable spectral quality between the DLPFC and OCC.

### 2.6 MRS data analysis

**Fig. 2:**
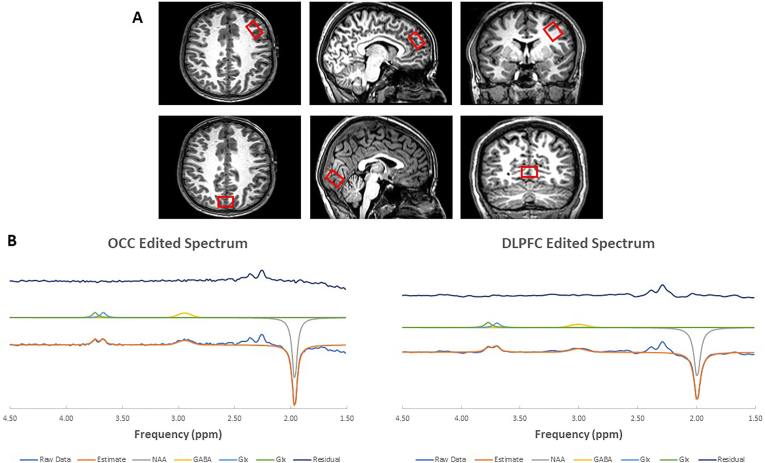
(A) Axial, sagittal and coronal images showing the placement of the voxels of interest and (B) Example spectra and AMARES fits from Dorsolateral Prefrontal Cortex (DLPFC) and Occipital lobe (OCC). The bottom trace shows the raw data and the full AMARES fit. The middle trace shows the individual components, while the top trace shows the residual after fitting. Note that the model does not include prior knowledge for glutamate, glutamine and GABA resonances between 2.2 and 2.5 ppm.

Quantification was conducted using the Advanced Magnetic Resonance (AMARES) (Vanhamme et al., 1997) routine in the Java-based magnetic resonance user's interface (jMRUI5.1, EU project) (Stefan et al., 2009). Individual transients were frequency-aligned, phase-corrected, and averaged within each acquisition session. Water unsuppressed spectra were acquired from the same locations and served as reference. No time-domain filtering was performed on the data before analysis by AMARES. We rejected MRS data from any subject in which there was a change in NAA line width of greater than 3 SD of the global mean linewidth before and after tDCS or sham stimulations such an effect could indicate movement between the two acquisitions. Metabolite concentrations including GABA+, glutamate + glutamine (Glx) and N-acetylaspartate (NAA) were calculated using the unsuppressed water signal from the same voxel as a concentration reference (only GABA + reported here), without performing a correction for voxel tissue composition. Tissue correction was not considered necessary since the same voxels are interrogated before and after stimulation or sham. In order to perform correlation analysis of GABA + across subjects, which could be affected by tissue composition differences, MRS voxels were segmented from the T1-weighted anatomical images into grey matter (GM), white matter (WM) and cerebrospinal fluid (CSF) using SPM8 (http://www.fil.ion.ucl.ac.uk/spm/). Voxel registration was performed using custom-made scripts developed in MATLAB by Dr. Nia Goulden, which can be accessed at http://biu.bangor.ac.uk/projects.php.en. The scripts generated a mask for voxel location by combining location information from the Philips SPAR file with orientation and location information contained within the T1 image. Voxel locations, example spectra and AMARES (Vanhamme et al., 1997) fits are illustrated in Fig. 2.

Spectra quality were assessed and artefactual spectra were removed from further analysis, as reported in Garg et al. (2022) and summarised in Table 1. The calculation of partial volume within the VOIs provided the percentage of each tissue type within each voxel (Table 1). There were no significant differences between the percentage of tissue fraction pre-and post-stimulation in any of the voxels. GABA+ was corrected for tissues fraction (GABA/(grey matter + white matter)) for the correlation analyses with effective connectivity.

**Table 1:** A summary of removal of spectra from the analysis. Spectra were rejected due to spectroscopic artefacts (e.g. poor water suppression, lipid contamination or broad line widths), > 3SD difference in pre-post intervention NAA line width, and acquisition difficulties. Tissue fraction refers to grey matter + white matter tissues.

		Reason for removal			
Session	Spectra location	Spectroscopic artefacts	NAA line width	Acquisition difficulties	Remaining number of spectra
Pre-tDCS	dlPFC	1	–	–	28
	OCC	–	–	–	29
Post-tDCS	dlPFC	3	2	–	24
	OCC	–	–	1	28
Pre-sham	dlPFC	–	–	–	31
	OCC	–	–	2	29
Post-sham	dlPFC	5	1		25
	OCC	2	1	3	25

### 2.7 Functional MRI acquisition

Imaging was performed on a 3 T Philips Achieva scanner using a 32-channel head coil with a SENSE factor 2.5. To maximise signal-to-noise (SNR), we utilised a dual-echo fMRI protocol developed by Halai et al. (2014). The fMRI sequence included 36 slices, 64 × 64 matrix, field of view (FOV) 224 × 126 × 224 mm, in-plane resolution 2.5 × 2.5 mm, slice thickness 3.5 mm, TR = 2.5 s, TE = 12 ms and 35 ms. The total number of volumes collected for each fMRI session was 144.

### 2.8 fMRI processing

Within the present work, we only analyse the fMRI data acquired during pre-stimulation and post-stimulation sessions. Image processing was done using SPM12 (Wellcome Department of Imaging Neuroscience, London; http://www.fil.ion.ucl.ac.uk/spm) and MATLAB R2023a. Dual echo images were extracted and averaged using in-house MATLAB code developed by Halai et al. (2014) (DEToolbox). First, functional images were slice time corrected and realigned to first image. Then, the short and long echo times were averaged for each timepoint. The orientation and location of origin point of every anatomical T1 image was checked and corrected where needed. Mean functional EPI image was co-registered to the structural (T1) image. Motion parameters estimated during co-registration of short echo-time images were input to Artifact Detection Tools (ART; https://www.nitrc.org/projects/artifact_detect/) toolbox along with combined dual echo scans for identification of outlier and motion corrupted images across the complete scan. The outlier detection threshold was set to changes in global signal 3 z-scores away from mean global brain activation. Motion threshold for identifying scans to be censored was set to 3 mm. Outlier images and images corrupted by motion were censored during the analysis by using the outlier volume regressors. Participants with less than 80 % of scans remaining were removed from analysis. Following the removal of participants with high motion and acquisition artefacts, 18 NF1 patients remained. Unified segmentation was conducted to identify grey matter, white matter, and cerebrospinal fluid. Normalisation to MNI space was done with diffeomorphic anatomical registration using exponentiated lie-algebra (DARTEL) (Ashburner, 2007) registration method for fMRI. Normalised images were interpolated to isotropic 2 × 2 × 2 mm voxel resolution. A 6x6x6mm full width at half maximum (FWHM) Gaussian smoothing kernel was applied.

### 2.9 Volumes of interest

To select volumes of interest (VOIs), we considered regions that have been previously involved with N-back task performance in adults (Owen et al., 2005) and adolescents (Andre et al., 2016). In addition, during selection of VOIs we considered regions affected by working memory in this dataset. To identify these regions, first-level mass univariate analysis using the General Linear Model based on the canonical haemodynamic response function. A high-pass filter with a cut-off at 128 s was applied to remove slow signal drifts. An autoregressive model of order 1 was fitted to estimate and remove serial correlations (Friston et al., 1995). The outlier volume censoring regressors and 6 motion parameters were included as covariates. An F-contrast for the main effect of the N-back task and a t-contrast for the effect of working memory (2-back > 0-back) were defined. Finally, to discover the regions that show effect of working memory, we performed four separate group-level analysis for pre-tDCS, post-tDCS, pre-sham and post-sham sessions. Age and sex were input as covariates.

**Fig. 3:**
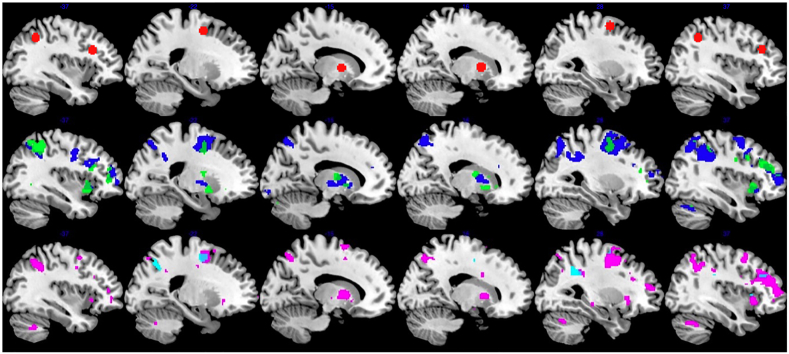
The red spheres signify the location of 8 VOIs selected for the analysis. Clusters signify the uncorrected p < 0.001 results of mass univariate 2-back>0-back contrasts performed separately for pre-atDCS (blue), post-atDCS (green), pre-sham (magenta) and post-sham (cyan) sessions.

Eight VOIs were defined as spheres with 6 mm radius (Fig. 3). The centres of the spheres were in bilateral Inferior Parietal Gyrus (IPG, MNI: 36 -52 43 and 36–52 43), bilateral Inferior Frontal Gyrus pars triangularis/dorsolateral Prefrontal Cortex (dlPFC, MNI -40 22 27 and 41 31 28), bilateral Superior Frontal Gyrus (SFG, MNI: 22 -1 52 and 28 -2 58), bilateral globus pallidus (MNI: 15 1 4 and 15 5 5). The first eigenvariate of the timeseries of all voxels in each VOI was extracted and adjusted by F-contrast for main effects (removing effects of motion and global signal artefacts). These signals were further used for DCM modelling and analysis.

### 2.10 Dynamic causal modelling (DCM)

DCM is a generative modelling approach which describes modulating effects of experimental manipulations on effective connectivity (Friston et al., 2003). DCMs include average and modulatory connectivity, respectively represented by A-matrix and B-matrix. Average connectivity refers to the average connections between brain regions, independent of task conditions. Modulatory connectivity refers to the impact of experimental inputs on connectivity between regions. The off-diagonal elements of the A-matrix represent the average extrinsic (inter-regional) connection strengths, and the diagonal elements represent the intrinsic (intra-region) inhibitory connection strengths. Meanwhile, the off-diagonal and diagonal elements of the B-matrix represent the modulatory strengths of the experimental manipulation on the average extrinsic and intrinsic connections.

#### 2.10.1 First-level DCM

**Fig. 4:**
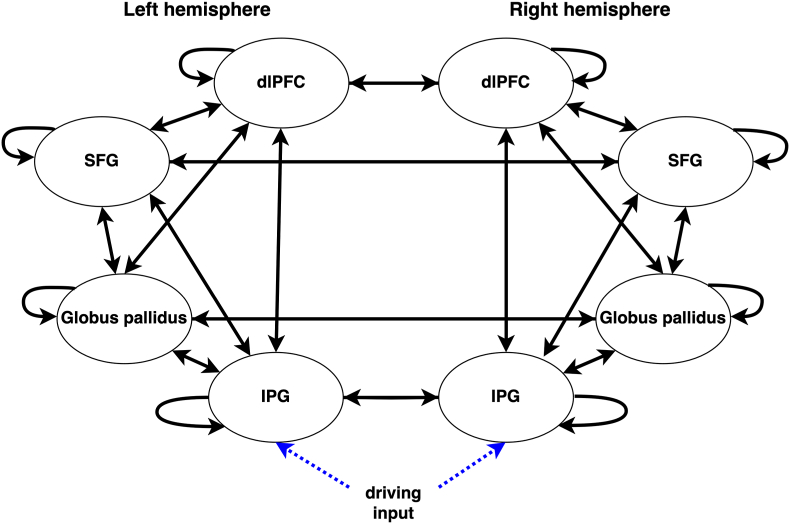
Graph representing the structure of the full DCMs used.

We estimated the effective connectivity for each subject with fMRI DCM (Friston et al., 2003). Separate DCMs were constructed and inverted for each session (pre-tDCS, post-tDCS, pre-sham, post-sham). Average connectivity was obtained across all N-back trials in each session. Modulatory effect of working memory was modelled by coding 2-back and 0-back with 1 and -1 respectively. The structure of the full DCMs used is shown in Fig. 4. Driving inputs entered the IPG of the two hemispheres (Ma et al., 2011).

#### 2.10.2 Second-level parametric empirical bayes

A second level-analysis was performed using the parametric empirical Bayes (PEB) approach to identify group level changes in connectivity as result of tDCS stimulation (Friston et al., 2016; Zeidman et al., 2019). This model included the mean connectivity, main effect of session, main effect of stimulation type and interaction between session and stimulation type, as well as estimating the between-subject variability. A Bayesian model comparison (BMC) approach was then used to test the hypothesis that interaction between session and stimulation type would manifest in intrinsic and extrinsic connectivity from dlPFC. To do this a model space was constructed by sequentially “switching off” the intrinsic and extrinsic connections originating from dlPFC, producing a total of 16 DCMs. Model comparison between all DCMs in the model space was then carried out based on their model evidences, which were calculated using Bayesian Model Reduction (BMR) (Friston et al., 2016). For model comparison, the model evidence was interpreted according to the scales proposed by Kass and Raftery (1995).

Finaly, Bayesian model averaging (BMA) was performed to obtain posterior parameter estimates while accounting for model uncertainty. In brief, BMA is an average of the DCM parameter values estimated under each model in the model space, weighted by the posterior probability of each model. These parameters represent the effect sizes of our experimental manipulation (tDCS) on connectivity parameters (Friston et al., 2016).

### 2.11 Relationship between effective connectivity and GABA+ and behavioural performance

For details on the effect of tDCS on GABA+ in the dlPFC and OCC and on behavioural performance, please refer to Garg et al. (2022). In the present work, Pearson's correlation analyses were performed to relate how DCM parameters relate to GABA+ from dlPFC only, since no changes related to tDCS were found for OCC.

Next, Pearson's correlation analyses were performed to relate how DCM parameters relate to behavioural performance. During correlation analysis between average connectivity parameters and behaviour, the average RT and IES for the 0-back and 2-back conditions were obtained per participant, per session. Accuracy was not correlated to average connectivity due to ceiling effects during 0-back condition. During correlation analysis between modulatory connectivity parameters and behaviour, the 2-back accuracy, RT and IES were analysed. In all these analyses, we used a liberal 0.05 alpha for the significance threshold.

## 3 Results

### 3.1 Session and stimulation effects on effective connectivity

**Fig. 5:**
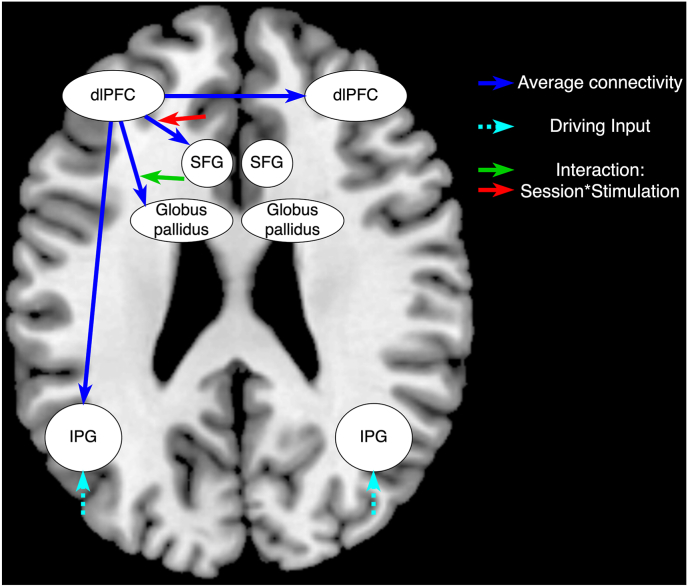
The results of BMA of the BMC winning model. The blue arrows represent the extrinsic excitatory average connectivity from dlPFC; and the green and red arrows represent respectively the positive and negative effect of the interaction between session and stimulation on the extrinsic connections.

The PEB and BMC methods were used to infer group-level effects of session and stimulation on the average connectivity of 0-back and 2-back conditions. The winning model according to BMC had a posterior probability (Ppost) of 0.33. There was very strong evidence of a positive effect of session on excitatory connections from the left dlPFC to the ipsilateral IPG (Ppost = 0.96), SFG (Ppost = 1) and right dlPFC (Ppost = 1). There was no notable evidence of an effect of session on the connection from dlPFC to the left globus pallidus (Ppost = 0.19). However, there was positive evidence of a positive effect of the interaction between session and stimulation on the excitatory connectivity from left dlPFC to left globus pallidus (Ppost = 0.82), and a negative effect on the excitatory connectivity from dlPFC to the left SFG (Ppost = 0.71). A schematic illustration of these results is presented in Fig. 5.

PEB and BMC were also used to infer the group level effects of session and stimulation on the modulation strength of working memory load. The null model in which all modulatory strength parameters were turned off was favoured by Bayesian model comparison (Ppost = 0.4), which indicated no effect of session or stimulation on connectivity modulation by working memory load.

### 3.2 Associations between effective connectivity and GABA+

Following the results of BMC, correlation analyses focused on investigating whether there is any association between GABA+ and left dlPFC's intrinsic connectivity or its extrinsic connectivity to globus pallidus and SFG (Table 2). There was a significant negative correlation between GABA+ and average intrinsic connectivity of dlPFC during the post-tDCS session (R = −0.65, p = 0.009), such that lower inhibitory intrinsic connectivity was associated with less GABA+. No other associations were found between dlPFC's connectivity parameters and GABA+.

**Table 2:** The results of correlation analysis between GABA+ and connectivity parameters. Bold font was used to highlight significant results (*p <* 0.05*)*, dashes were placed where no analysis was performed, since there was correlation in either pre-tDCS or post-tDCS session.

	pre-tDCS		post-tDCS		pre- to post-tDCS change	
	R	*p*	R	*p*	R	*p*
**Average connectivity**						
dlPFC to self	−0.25	0.331	**−0.65**	**0.009**	0.27	0.335
left dlPFC to left globus pallidus	0.14	0.594	0.26	0.349	–	–
left dlPFC to left SFG	0.1	0.695	0.18	0.515	–	–

**Modulatory connectivity**						
dlPFC to self	0.05	0.839	−0.02	0.932	–	–

### 3.3 Associations between effective connectivity and behavioural performance

Correlation analyses of average and modulatory pre- and post-tDCS connectivity parameters and behavioural performance revealed no linear correlations between behavioural performance and intrinsic connectivity of left dlPFC or connectivity from left dlPFC to globus pallidus or SFG. Table 3 summarises significant correlations between connectivity parameters and behaviour, and Supplementary Material 1 summarises all performed correlations between connectivity parameters and behaviour.

**Table 3:** The significant results (*p <* 0.05*)* of correlation analysis between behavioural performance and connectivity parameters. Dashes were placed where results were not significant.

		Average connectivity				Modulatory connectivity			
		pre-active		post-active		pre-active		post-active	
		R	*p*	R	*p*	R	*p*	R	*p*
**IES**									
	left globus pallidus to self	−0.52	0.027	–	–	–	–	–	–
	left IPG to self	−0.61	0.008	–	–	–	–	–	–
	left to right globus pallidus	0.59	0.011	–	–	–	–	–	–
	right to left globus pallidus	0.54	0.021	–	–	–	–	–	–
	right IPG to right globus pallidus	0.48	0.042	–	–	–	–	–	–
	right IPG to self	–	–	−0.59	0.009	–	–	–	–
	left SFG to self	–	–	–	–	−0.73	0.001	–	–
	left to right SFG	–	–	–	–	0.56	0.017	–	–
	left to right IPG	–	–	–	–	–	–	0.047	0.049
**RT**									
	right IPG to globus pallidus	−0.52	0.026	–	–	–	–	–	–
	right IPG to self	–	–	0.51	0.032	–	–	–	–
	left SFG to self	–	–	–	–	−0.78	<0.001	–	–
	left to right SFG	–	–	–	–	0.53	0.025	–	–

## 4 Discussion

In this paper, we analysed effective connectivity to investigate the effect of tDCS stimulation on neural correlates of working memory processes. The key finding of this work was that administration of tDCS to dlPFC resulted with reduced connectivity between itself and frontal lobes, coupled with increased connectivity to subcortical structures. This suggests a shift in the functional dynamics of the brain regions involved in working memory and attention. We also discovered neurochemical associations with these changes in the form of an association between GABA+ and sensitivity of left dlPFC to inputs, where reduced self-connectivity of this region was associated with lower GABA+. However, connectivity parameters of dlPFC showed no association with performance on an N-back task. This work offers novel insight to complex interactions between neurostimulation, neural connectivity, and neurochemical processes in the brain. These findings contribute to a better understanding of the role of the left dlPFC in cognitive control functions and open new avenues for research in NIBS and its clinical applications.

This work supplements Garg et al. (2022) with novel analysis of effective connectivity changes associated with administration of sham and anodal tDCS. Here, we uncovered the effects of administration of tDCS that persisted during the post-tDCS session. Specifically, we found reduced the average connectivity from left dlPFC to left SFG and increased connectivity from left dlPFC to left globus pallidus. This finding likely reflects changes to the neural substrates of cognitive processing conducted during N-back task performance. The left frontal regions are implicated in executive control processes through inhibitory control of responses (Swick et al., 2008), anticipatory attention (Solbakk and Løvstad, 2014), maintenance of working memory (Rypma et al., 1999), and goal-directed behaviour (Arimura et al., 2013). Importantly, the left frontal regions have widely been related to error monitoring, interference resolution and selection during retrieval (Fitzgerald et al., 2010; Nelson et al., 2009; Öztekin et al., 2009), particularly but not only in the verbal domain (Zhang et al., 2004). Therefore, tDCS has likely enhanced excitatory signalling of top-down control and interference resolution of competing stimuli from dlPFC to globus pallidus (Taylor et al., 2007). Overall, this may result with increased stimulus filtering of relevant stimuli prior to relay of stimuli to working memory processes conducted within the parietal regions (Baier et al., 2010), or prior to relay of appropriate motor response selection to the sensorimotor cortices (Jaeger and Kita, 2011). This is an important finding for the NF1 population, because many NF1 patients suffer from increased distractibility and decreased filtering of irrelevant information (Rowbotham et al., 2009), and as many as 50 % of individuals with NF1 receive comorbid diagnosis of Attention Deficit Hyperactivity Disorder that is characterised by difficulty in information filtering (Garg et al., 2012, 2013). Therefore, the findings suggest that tDCS could potentially serve as a therapeutic tool to address inattention-related symptoms by enhancing the brain's ability to filter information and improve attentional control. Overall, understanding the effects of these effective connectivity changes on control processes offers important future directions for NIBS research in NF1. Specifically, investigating the specific aspects of cognitive control (e.g., stimulus monitoring, stimulus filtering, sustained attention, selection of motor responses) and specific parameters of tDCS (e.g., intensity, duration, frequency) that yield optimal cognitive benefits for patients could refine treatment protocols.

Further, Garg et al. (2022) demonstrated that application of tDCS was associated with reduction of GABA+. Here we additionally found that lower inhibitory intrinsic connectivity of left dlPFC was associated with less GABA + post-tDCS, which indicates an increased dlPFC's excitability during the N-back task post-tDCS. This result suggests that by reducing GABA+, tDCS might make dlPFC more sensitive and responsive to external stimulation. This can affect how the dlPFC processes information, potentially enhancing its ability to engage in working memory performance, attention control, and cognitive flexibility the N-back task. Future research should explore this by investigating how tDCS impacts how regulation of intrinsic connectivity during tasks is related to GABA+ (Clark et al., 2011; Jocham et al., 2012; Krause et al., 2013), and its ratio to glutamate (Al-Otaish et al., 2018; Krause et al., 2013).

There are several important methodological considerations for interpreting the results of this work. First, the DCM method demonstrated the modulatory effect of tDCS stimulation on how dlPFC influences or drives the activity of other regions. The results show a reorganisation of these causal interactions. Importantly, we are not testing the direct effect of the stimulation on the local activity of regions outside the stimulation site. This will be an important consideration for future research, because other work demonstrates that the tDCS electrode configuration can change effects of stimulation (i.e. magnitude and location of effects) (Kuo et al., 2013), particularly depending on current intensity, electrode size, and electrode placement (Batsikadze et al., 2013; Bikson et al., 2010; Saturnino et al., 2015). For this reason, it will be important for future research to carefully explore how the tDCS setup influences neuronal interactions of the dlPFC and beyond in this patient group. Further, the field of view of the T1 images was too narrow to calculate electric field magnitude induced by tDCS in all areas of the brain. It is possible that variations in surrounding skull and tissue thickness might have led to differences in the magnitude of the field received by participants. Having these estimates could enable additional analyses, such as exploring how local inhibition and other effective connectivity parameters relate to the estimated field strength. As a result, the current findings remain focused on assessing changes in effective connectivity due to stimulation.

This study faces several limitations that should be acknowledged. First, patients were not taken off their regular medication, which may affect their cognitive performance, the effects of this were not accounted for. Second, DCM BMR did not allow us to test whether the interaction between session and stimulation would be seen in the average intrinsic connectivity of left dlPFC. Instead, DCM BMR always includes all intrinsic connections in the average connectivity. Therefore, we only formally explored the nested models of the effects of tDCS on extrinsic connectivity from left dlPFC. Further, In this work, we focused on connectivity of dlPFC to other regions related to working memory, to understand if stimulation has affected how dlPFC may drive activity of other areas. It will also be useful to understand how stimulation of basal ganglia, achieved with other electrode configurations, may impact subcortical signalling during working memory (Bunai et al., 2021). Further, our study is limited by a lack of extensive analysis of psychometrics of attentional capacities in this sample. Therefore, our proposed association between changes to top-down attentional control processes and changes in connectivity and behaviour from pre-tDCS to post-tDCS is speculative. Complementary attentional and linguistic mechanisms in NF1 must continue to be explored to understand the effects of tDCS. We cannot comment on the reliability of long-term effects of tDCS stimulation, and the effects we observed here may be transient.

In summary, this study revealed that tDCS decreased average excitatory connectivity from the left dlPFC to the left SFG and increased connectivity to the left globus pallidus in adolescents with NF1. We suggest that these changes likely reflect enhanced top-down control and filtering of relevant information. Future research should further explore the relationship between attentional processes and neural connectivity in NF1, as well as the long-term effects of tDCS. This study provides novel insights into the neural mechanisms underlying tDCS effects and highlights potential avenues for improving cognitive interventions in NF1 patients.

## CRediT authorship contribution statement

**Marta Czime Litwińczuk:** Data curation, Formal analysis, Visualization, Writing – original draft. **Shruti Garg:** Conceptualization, Data curation, Formal analysis, Funding acquisition, Methodology, Project administration, Writing – review & editing. **Stephen R. Williams:** Formal analysis, Methodology, Visualization, Writing – review & editing. **Jonathan Green:** Funding acquisition. **Caroline Lea-Carnall:** Conceptualization, Methodology, Project administration, Supervision, Writing – review & editing. **Nelson J. Trujillo-Barreto:** Conceptualization, Methodology, Supervision, Writing – review & editing.

## Data/code availability

The patient data have been deposited on the Sage Bionetworks data repository https://www.synapse.org/Approved researchers can request to obtain the data which are subject to data sharing agreements. Codes for data processing and analysis are available at https://github.com/MCLit/NF1-DCM-WM

## Ethics statement

Ethics approval for the study was obtained from the North West-Greater Manchester South Research Ethics Committee (reference: 18/NW/0762). Written informed consent was obtained from the parents and older adolescent participants and assent was obtained from the younger participants.

## Declaration of competing interest

The authors declare the following financial interests/personal relationships which may be considered as potential competing interests: Shruti Garg, Jonathan Green and Caroline Lea-Carnall reports financial support was provided by 10.13039/100014653NIHR Manchester Biomedical Research Centre. Marta Czime Litwinczuk reports financial support was provided by 10.13039/100000025National Institute of Mental Health Division of Translational Research. Shruti Garg reports financial support was provided by 10.13039/100014804Neurofibromatosis Therapeutic Acceleration Program. Jonathan Green reports financial support was provided by 10.13039/501100000272NIHR Senior Investigator Award. Nelson J Trujillo-Barreto reports financial support was provided by 10.13039/501100000265Medical Research Council. If there are other authors, they declare that they have no known competing financial interests or personal relationships that could have appeared to influence the work reported in this paper.

## Data Availability

Manuscript and Title page detail the data and code locations
